# The telovelar approach for fourth ventricular tumors in children: is removal of the posterior arch of C1 necessary?

**DOI:** 10.1007/s00381-024-06443-3

**Published:** 2024-05-04

**Authors:** Anna Cho, Maria Aliotti Lippolis, Johannes Herta, Muhammet Dogan, Cora Hedrich, Amedeo A. Azizi, Andreas Peyrl, Johannes Gojo, Thomas Czech, Christian Dorfer

**Affiliations:** 1 Department of Neurosurgery, Medical University of Vienna, Waehringer Guertel 18-20, 1090 Vienna, Austria; 2 Department of Pediatrics and Adolescent Medicine, Comprehensive Center for Pediatrics and Comprehensive Cancer Center, Medical University of Vienna, Vienna, Austria

**Keywords:** Telovelar approach, Pediatric brain tumor, Fourth ventricular tumor

## Abstract

**Purpose:**

Various surgical nuances of the telovelar approach have been suggested. The necessity of removing the posterior arch of C1 to accomplish optimal tumor exposure is still debated. Therefore, we report on our experience and technical details of the fourth ventricular tumor resection in a modified prone position without systematic removal of the posterior arch of C1.

**Methods:**

A retrospective analysis of all pediatric patients, who underwent a fourth ventricular tumor resection in the modified prone position between 2012 and 2021, was performed.

**Results:**

We identified 40 patients with a median age of 6 years and a M:F ratio of 25:15. A telovelar approach was performed in all cases. In 39/40 patients, the posterior arch of C1 was not removed. In the remaining patient, the reason for removing C1 was tumor extension below the level of C2 with ventral extension. Gross or near total resection could be achieved in 34/39 patients, and subtotal resection in 5/39 patients. In none of the patients, a limited exposure, sight of view, or range of motion caused by the posterior arch of C1 was encountered, necessitating an unplanned removal of the posterior arch of C1. Importantly, in none of the cases, the surgeon had the impression of a limited sight of view to the most rostral parts of the fourth ventricle, which necessitated a vermian incision.

**Conclusion:**

A telovelar approach without the removal of the posterior arch of C1 allows for an optimal exposure of the fourth ventricle provided that critical nuances in patient positioning are considered.

## Introduction

In pediatric neurosurgery, resection of tumors within the fourth ventricle is a common scenario, and pediatric neurosurgeons tend to be more familiar with the anatomical and surgical details needed to gain safe access to the fourth ventricle than general neurosurgeons. To achieve the most atraumatic surgical approach as possible, patient positioning is fundamental to allow for an adequate exposure [1–3]. In general, patients may be positioned in prone or semi-sitting positions, each of which is considered to have advantages, disadvantages, and limitations [2–5].

In addition to patients’ positioning, the surgical approach itself may also vary, depending on the surgeons’ preference. One of the two main surgical corridors to fourth ventricular tumors is the transvermian approach, allowing a wide operative exposure [6]. However, due to various complications of the transvermian approach, especially for tumors without vermian infiltration, such as the posterior fossa syndrome including cerebellar mutism, the vermian sparing telovelar approach, which is taking advantage of a natural corridor, has increasingly gained acceptance [6, 7]. Additional advantages of this surgical corridor are the favorable exposure to the superolateral and lateral recesses with the foramina of Luschka [6]. Since its first description, various surgical nuances of the telovelar approach have been suggested and sometimes misleadingly discussed when it comes to tumors located in the lower vermis [7, 8]. One of these surgical details is the removal of the posterior arch of C1 to accomplish a more extended exposure of the most rostral parts of the fourth ventricle. While many neurosurgeons tend to remove the posterior arch of C1 because (i) it is easy and (ii) they have the impression that it is beneficial in terms of exposure, the discussion in the literature on this surgical nuance has been scarce [7, 9]. Following the detailed comparative anatomical analysis of Deshmukh et al., who concluded that removal of the posterior arch of C1 is necessary in order to achieve the widest exposure, no article has addressed this notion [9].

Therefore, we sought to present our experience with resection of fourth ventricular tumors focusing on the necessity of removal of the posterior arch of C1. We report on our surgical nuances aiming at an optimal intraoperative exposure via a telovelar or telovelar-like approach allowing for a maximal safe resection and being as atraumatic as possible including the preservation of the posterior arch of C1.

## Methods

### Study population and patient characteristics

A retrospective analysis of all pediatric patients, who were operated for a posterior fossa tumor between 2012 and 2021, was performed. The inclusion criteria for this study were age < 18 years and neurosurgical resection of a newly diagnosed fourth ventricular tumor through a telovelar approach by the two experienced pediatric neurosurgeons T.C. and C.D. For medulloblastomas originating from or infiltrating the most caudal parts of the vermis (nodulus, uvula), dissection of the tonsillo-vermian cleft to maximize tumor exposure was performed in addition to resection of the infiltrated vermis. We refer to this as the telovelar-like approach. At our institution, the modified prone position for the telovelar and telovelar-like approach is the preferred method for the resection of fourth ventricular tumors [10].

**Fig. 1:**
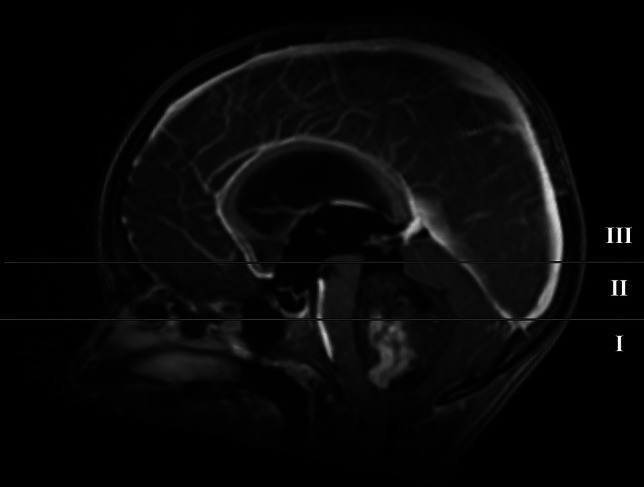
Tumor localization. (I) Below the fastigium, (II) below the velum, and (III) within the aqueduct

Detailed patient characteristics are displayed in Table 1. Tumors were categorized based on their extension on the sagittal plane of T1-weighted MRI as follows: (I) below the fastigium, (II) below the velum, (III) within the aqueduct (Fig. 1).

**Table 1:** Patient characteristics. The table demonstrates the detailed patient characteristics of the study population (*n* = 40). All patients underwent a fourth ventricle tumor resection, positioned in our modified prone position. Tumors were categorized based on their extension on the sagittal plane of T1-weighted MRI

	**Number of patients (%)**
**Age**, in years, median (range)	6 (1–16)
**Sex**	
Male	25 (63%)
Female	15 (37%)
**Symptoms of increased intracranial pressure**	
Yes	36 (90%)
No	4 (10%)
**Tumor localization**	
Below the fastigium	25 (63%)
Below the velum	14 (35%)
Within the aqueduct	1 (2%)
**Treatment for acute decompensated hydrocephalus**	
Yes	27 (68%)
Extraventricular drainage	26/27 (96%)
Endoscopic ventriculostomy	1/27 (4%)
No	13 (32%)
**Removal of the posterior arch of C1**	
Yes	1 (2%)
No	39 (98%)

### The modified prone position for the telovelar and telovelar-like approach

**Fig. 2:**
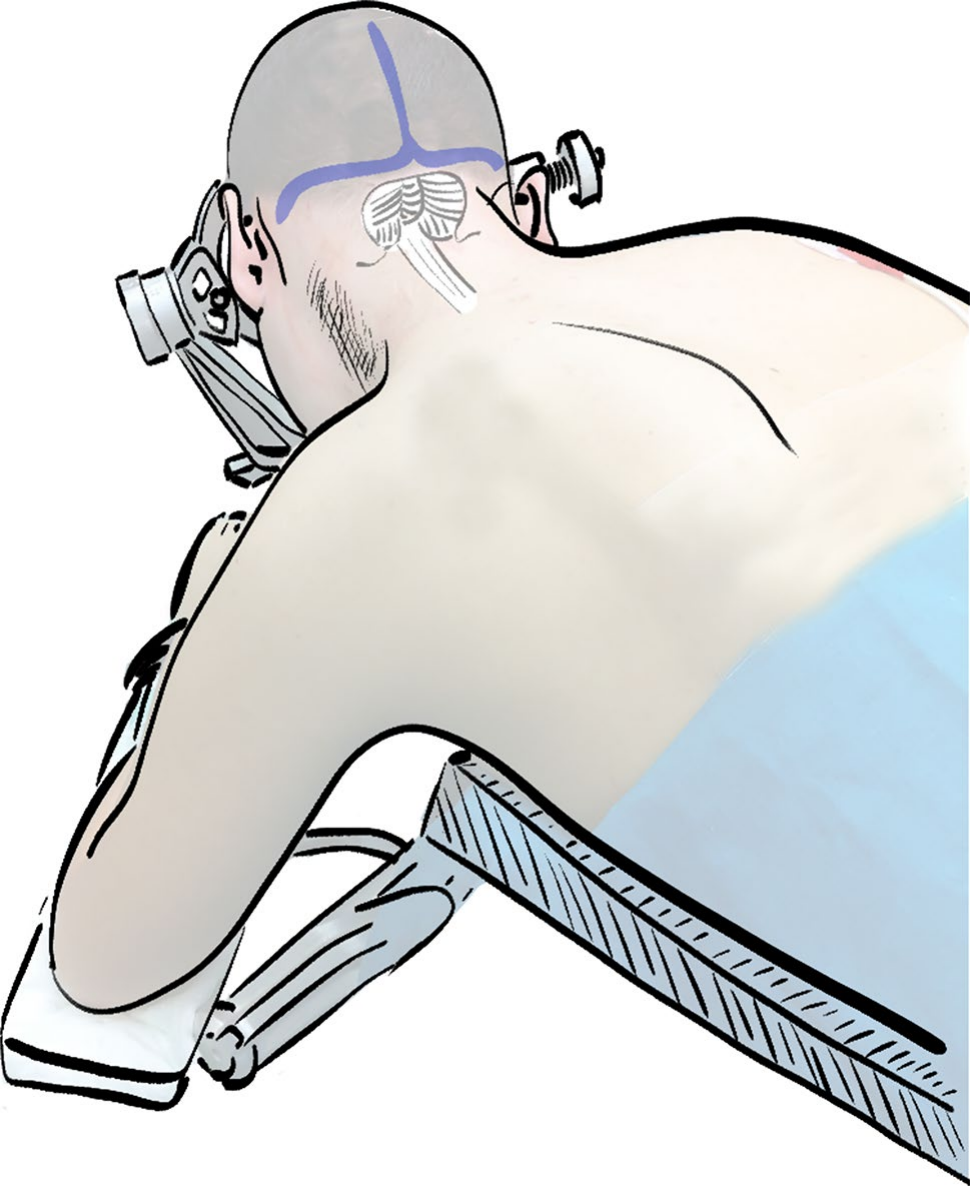
Illustration of the modified prone position

First, the patient is brought into a prone position with the aim of getting the patient’s body as far left on the table as possible. The surgeon is sitting on the left side of the patient instead of standing or sitting in front of the patient. This enables elevation of the head above heart level and at the same time the necessary amount of flexion to guarantee a steep angle of view. Depending on the individual patient´s body physique and the width of the shoulders, the ipsilateral arm is either extended over the edge of the table so that the surgeon comes closer to the operative field or in small children, this arm may be left next to the torso. To further increase the surgeon’s comfort, the head of the patient is tilted to the contralateral side so that the midline comes parallel to the surgeon’s view. For better demonstration, Fig. 2 illustrates the surgeon’s view of our proposed modified prone position.

## Results

We identified 40 pediatric patients with newly diagnosed fourth ventricular tumors, who underwent a microsurgical resection through the telovelar (*n* = 23) or telovelar-like (*n* = 17) approach in our modified prone position.

At the time of diagnosis, the median age was 6 years (range 1–16), and the M:F ratio was 25:15. The majority of patients (36/40, 90%) presented with signs and symptoms of increased intracranial pressure. In 27/40 (68%) patients, placement of extraventricular drainage (*n* = 26) or endoscopic ventriculostomy (*n* = 1) was performed before tumor resection.

The most common tumor extension on the sagittal plane of T1-weighted MRI was category I (below the fastigium, 63%), followed by category II (below the velum, 35%) and category III (within the aqueduct, 2%).

In 39/40 (98%) of the cases, the posterior arch of C1 was not removed. In the remaining patient, the reason for removing the posterior arch of C1 was tumor extension below the level of C2 with ventral extension. The histopathological report of this patient revealed an ependymoma.

Gross total resection or near total resection could be achieved in 34/39 (87%), and subtotal resection in 5/39 (13%) patients. In none of the patients, a limited exposure, sight of view, or range of motion caused by the posterior arch of C1 was encountered, necessitating an unplanned removal of the posterior arch of C1. Importantly, in none of the cases, the surgeon had the impression of a limited sight of view to the most rostral parts of the fourth ventricle, which necessitated a vermian incision.

The final histopathological reports of those 39 patients revealed 21 medulloblastomas, 13 pilocytic astrocytomas, and 5 ependymomas. The tumor histology did not influence the extent of tumor resection. Further details on tumor histology are shown in Table 2.

**Table 2:** Tumor characteristics. The table demonstrates the details on extent of resection and tumor histology of 39/40 (98%) patients who underwent a fourth ventricle tumor resection, positioned in our modified prone position, without removing the posterior arch of C1

	**Number of patients (%)**
**Extent of tumor resection**	
GTR/NTR	34 (87%)
STR	5 (13%)
**Intraoperative necessity of unplanned removal of C1**	
Yes	-
No	39 (100%)
**Limited view to rostral parts of the fourth ventricle**	
Yes	-
No	39 (100%)
**Tumor histology**	
Medulloblastoma	21 (54%)
WNT	3/21 (14%)
SHH	-
Group 3 or 4	17/21 (81%)
Unknown	1/21 (5%)
Pilocytic astrocytoma	13 (33%)
Ependymoma	5 (13%)

*GTR* gross total resection, *NTR* near total resection, *SHH* sonic hedgehog, *STR* subtotal resection, *WNT* wingless-related type

## Discussion

A good exposure of the upper fourth ventricle is critical when resecting tumors to minimize surgical trauma in this sensitive area. To achieve this, many nuances related to patient positioning, surgical route, and removal of the posterior arch of C1 have been suggested [3, 7, 9, 11]. In this study, we share our experience showing that neither removal of the posterior arch of C1 nor an extension of the vermian incision beyond the tumor-infiltrated part of the lower vermis is necessary to get an adequate overview of the upper fourth ventricle in a modified prone position.

So far, the resection of pediatric fourth ventricular tumors has been performed in a semi-sitting position by other neurosurgeons arguing that this is necessary to provide the steep angle of view for an adequate exposure [12]. The semi-sitting position, however, may come with potential complications that are generally not encountered in the prone position. Hence, if this benefit of the semi-sitting position is relativized by an optimal use of the possibilities in the prone position, the known drawbacks such as venous air embolism and hemodynamic instabilities may disadvantage this position [3]. Although both semi-sitting and prone positions for the resection of a posterior fossa tumor have been described innumerably, studies comparing both against each other, especially in the pediatric population, are still scarce [2, 5, 13]. In the study of Baro et al., the authors described that both positions can be considered safe. However, this study was limited by the number of patients (*n* = 30), operated in two different study centers, as well as the fact that only patients with pilocytic astrocytomas were included [2]. Beside the angle of view, proponents of the semi-sitting position also feel that the pressure in the posterior fossa is increased in the prone position through venous congestion, making tumor resections more traumatic. We agree that if patients are positioned completely flat and neutral, or even with the head flexed downwards (also known as Concorde position) below heart level, in order to get a steep angle of view, venous congestion leads to a full posterior fossa complicating tumor resections. For this reason, we suggest sitting next to the patient and taking advantage of an elevated upper body and head. At the same time, it opens the possibility of a downward flexion of the head to get a steep angle of view but remaining above heart level with the head. With these maneuvers, removal of the posterior arch of C1 is not necessary to get a good exposure of the upper fourth ventricle through a telovelar approach.

If the head is in a more neutral position, it seems obvious that, as many neurosurgeons argue, the removal of the posterior arch of C1 is necessary to get the range of view and motion needed for a safe tumor removal.

Alternatively, this range of motion and exposure of the upper fourth ventricle is accomplished by vigorously lifting up or incising the lower vermis, i.e., taking a more transvermian route to the tumor. It seems, however, generally accepted that the incision of non-infiltrated parts of the vermis increases the risk for a posterior fossa syndrome including cerebellar mutism. Thus, if taking advantage of an optimized positioning of the patient may contribute to reducing this risk, any attempt should be made.

At this point, one may argue that it does not matter whether or not the posterior arch of C1 is removed. To the best of our knowledge, there is no data given in the literature that would support a potential disadvantage of the removal of the posterior arch of C1 in children. However, it has to be questioned whether this justifies removing it if it is not necessary. This necessity, however, has scarcely been debated so far [7, 9]. Deshmukh et al. provided the most comprehensive study on whether or not the removal of the posterior arch of C1 offers an advantage in an anatomic study of six cadaver heads. With the help of a robotic microscope, the surgical exposure was calculated from triangles formed by defined anatomic points. They concluded that the removal of the posterior arch of C1 with the telovelar approach significantly increased the vertical angle of approach to the rostral aspect of the fourth ventricle and offered a larger working angle [9]. While we agree that from an anatomic point of view removal of the posterior arch of C1 would allow for a steeper angle of view by coming from more below, in clinical practice, this theoretical advantage is hindered by the spinal process of C2, nuchal musculature, and skin. The standard skin incision for a telovelar approach is usually not performed below C2/C3, neither is a subperiosteally dissection of the muscles from C2 or even C3. However, in order to take advantage of the theoretical increase in the angle of view by removal of the posterior arch of C1, these maneuvers would be necessary. This becomes clear when looking at Fig. 2A in their paper [9].

In our experience, the optimized use of the prone position combines the advantages of reducing the well-known risks of air embolism and pneumocephalus of the semi-sitting position and at the same time allows good exposure of the entire fourth ventricle through a telovelar approach obviating the need for removal of the posterior arch of C1, with a comfortable position of the surgeon.

One of our study limitations is the lack of any quantitative measures objectifying the surgeon’s impression of an adequate exposure. Thus, this report reflects the personal experience of the two experienced operating pediatric neurosurgeons in this series. Still, a debate on this specific aspect of the telovelar approach has not been conducted qualifying our large case series interesting and valuable.

## Conclusions

A telovelar approach without the removal of the posterior arch of C1 allows for an optimal exposure of the fourth ventricle provided that critical nuances in patient positioning are considered.

## Data Availability

No datasets were generated or analyzed during the current study.

## Abbreviations

GTR: Gross total resection
NTR: Near total resection
SHH: Sonic hedgehog
STR: Subtotal resection
WNT: Wingless-related type
